# Insight into the Effects of Drying Methods on Lanzhou Lily Rehydration

**DOI:** 10.3390/foods12091817

**Published:** 2023-04-27

**Authors:** Xinyu Zhang, Lu Xue, Zijian Wu, Wen Zhang, Han Zhang, Cuiyu Zhao, Dandan Liu

**Affiliations:** Tianjin Key Laboratory of Food Biotechnology, School of Biotechnology and Food Science, Tianjin University of Commerce (TJCU), Tianjin 300134, China

**Keywords:** dried lily scale, rehydration performance, microwave vacuum drying, lily starch, water absorption ability

## Abstract

This study investigated the effects of drying methods (hot air drying (HAD), microwave vacuum drying (MVD), and vacuum freeze drying (VFD)) on the rehydration performance (RP) of dried Lanzhou lily scales (LLS). Rehydration rate and water migration showed that MVD had the best RP, followed by VFD, while HAD had the worst. The results of additional morphology observation using scanning electron microscopy (SEM) and micro X-ray computed tomography (CT) imaging showed that both MVD and VFD created more channels in more porous structures, which facilitated their better RP than that by HAD. The results also revealed the spatial structure diversity (including pores, channels size, and internal network) of each dried Lanzhou lily scale group. In addition, studies analyzed how drying techniques affected the physiochemical properties of lily starch, including its water solubility, pasting profiles, and starch particle morphology. The findings indicated that when MVD was in operation, partial gelatinization in lily starch was brought about by thermal effects, allowing MVDS crystals to change from B-type to V-type and causing MVDS to have better water absorption ability. Consequently, despite the fact that MVD’s desiccated lilies have a lower porous structure and thinner channels than VFD’s, MVD has a higher RP than VFD.

## 1. Introduction

Lanzhou lily (*Lilium davidii* var. unicolor), a mutation variant of *L. davidii* Duchartre, belongs to the genus *Lilium* in the family *Liliaceae* [1], and its edible and sweet bulb is the only one used for both medicine and food up till now [2]. The semi-arid continental climate in Lanzhou Gansu province, including plentiful sunshine and excellent temperature variation between day and night, makes proper conditions for the photosynthesis and the subsequent accumulation of carbohydrates in the Lanzhou lily underground bulb [3]. In addition, Lanzhou lily bulb also contains various bioactive phytomolecules, including polyphenols, polysaccharides, saponins, and colchicines [4], making it possess a healthy function of antioxidation, anti-inflammation, relieving cough, anti-insomnia, and so on [5,6]. Starch is the essential form of carbohydrate in the bulb and constitutes approximately 53% to 69% of the lily bulb, based on dry weight, with the level of amylose ranging from 26 to 30% [7,8]. Physicochemical properties of lily starch are definitely associated with their applications in the food and nonfood industries and also in turn could easily be affected by food processing, which further influences the sensory quality of the lily final product [8,9].

The Lanzhou lily bulb harvesting season is typically brief (August to October each year), and the high ambient temperature and water content make the cumulative newly-collected bulbs susceptible to perishability and cause them to turn brown or violet-red [9], thereby diminishing the nutritional value and aesthetic quality of the final product. Therefore, a good method for safe and long-term preservation is to make fresh bulbs into dried ones [10]. Traditionally, natural air/sun drying and hot air drying (HAD) are used for lily bulbs, but both of them have great disadvantages [11]. Long-term external exposure of the former causes pollution and oxidation, and long processing time and high treatment temperature of the latter cause browning and hardening of dried bulbs, resulting in a poor sensory experience for consumers. Worse yet, some outlaws choose sulfur fumigation to whiten brown bulbs, creating SO_2_ content that exceeds the standard, which would have an adverse impact on the consumer’s health. Vacuum freeze drying (VFD) is an established method, operated under the conditions of vacuum and low temperature, especially for rare or highly valuable herbs and foods [12,13], and it could well-preserve properties of food or initial raw material, including appearance, the content of thermo-sensitive components and biological activity, etc. [14]. Microwave vacuum drying (MVD) combines vacuum and microwave drying and has the benefits of each. It has outstanding performance in maintaining material quality and efficiency [15]. Furthermore, it is well-known that almost all dried food material ought to be rehydrated before consumption or processing, and dried Lanzhou lily bulbs are no exception. Rehydration capacity/rate should be one of the key evaluation indicators for choosing drying methods. It could also reflect the physical changes in dried foodstuff [16]. 

However, the effect of structural changes in lily starch on the water absorption and rehydration properties of dried lily scale samples has not been thoroughly investigated. This study aims to explain differences in the rehydration properties of lily scales treated with three different drying methods (HAD, MVD, and VFD). This work could provide a certain evaluation measure for finding efficient drying methods to produce dehydrated food material, especially dried lily scales.

## 2. Materials and Methods

### 2.1. Materials

Fresh Lanzhou lily (*Lilium davidii* var. unicolor) bulbs used in this study were purchased from Lanzhou city Gansu Province, and fresh lily bulb scale samples were selected for drying according to appearance, such as uniform size and whiteness, no mechanical deterioration, and no damage by disease and insect. All selected lily scales were thoroughly cleaned before drying. Chemical reagents used were of analytical purity grade, and the deionized water was obtained by an ultrapure water system (Arium-mini Plus; Sartorius, Göttingen, Germany).

### 2.2. Drying Methods

These cleaned and selected lily bulbs were divided into 3 groups and subjected to hot air drying (HAD), vacuum freeze drying (VFD), and microwave vacuum drying (MVD), and obtained dried lily bulb scale samples were named as: HAD, MVD, and VFD, respectively.

The HAD sample was placed in an oven with hot air (CT-C Series Hot Air Circulation Oven, Changzhou Jukai Drying Equipment Co., Ltd., Changzhou, Jiangsu, China) and dried at 80 °C for 4–6 h (rotation every hour) until its ultimate moisture content reached 5.06 ± 0.05% [11]. Samples of the VFD group were pre-frozen in a blast freezer (MDC-XBL-BL-BE3, Dongguan Huadao Energy Saving Technology Co., Ltd., Dongguan, Guangdong, China) at −30 °C for 3–4 h in sample holding trays (loading density 3.52 kg/m^2^) and dried for 72 h in a pilot-scale freeze-drier (LGJ-20F, Beijing Songyuanhuaxing Technology Develop Co., Ltd., Beijing, China) under vacuum (100–300 Pa), with plate temperature being 40 °C and condenser temperature being −50 °C; the final moisture content of samples reached 4.72 ± 0.06% [12]. Samples of the MVD group were dried (HWZ-30B, Gansu Tianshui Huayuan Pharmaceutical Equipment Technology Co., Ltd., Tianshui, Gansu, China) under vacuum (−0.09 MPa) at 45~55 °C for around 55 min, with microwave power being 700 W, rotation speed being 6 r/min, and the final moisture content came to 4.86 ± 0.13% [14].

### 2.3. Rehydration Performance Evaluation

Rehydration performance of dried lily bulb scales was conducted according to the method of Wang [17]. About 3.0 g of dried samples were accommodated in a 50-mL ultra-filtration centrifuge tube containing 35 mL distilled water and then stayed at 50 °C in thermostatic circulating water bath (ICC basic, IKA Ltd., Staufen, Germany). At the fixed interval (0, 5, 10, 15, 20, 25, 30, 40, 50, 60, 80, 100 and 120 min), excess water was removed by centrifugation (at 3000× *g* for 10 min), followed by further blotting with paper towels, and subsequently lily bulb scale samples were taken out to take photographs and then were weighed to calculate the rehydration ratio (Equation (1)).

Rehydration ratio (g/g) = W_t_/W_0_ (1)

W_t_ is the weight of the sample at t minutes of rehydration; W_0_ is the dry weight of the sample before hydration.

The water absorption rate (WAR) of the lily bulb during rehydration was calculated by using Equation (2) [18]:

Water absorption rate (g/g.min^−1^) = (Rr_t2_ − Rr_t1_)/(t_2_ − t_1_) (2)

where Rr_t1_ and Rr_t2_ are the rehydration ratio at time t_1_ and t_2_; t (min) is the rehydration time.

### 2.4. Low Field Nuclear Magnetic Resonance (LF-NMR)

Each sample was kept in the air for 10 min before LF-NMR, and LF-NMR spectra was carried out using NMR Analyzer (Meso MR23-060H-I, Niumag Electric, Shanghai, China). Carr–Purcell–Meiboom–Gill (CPMG) pulse sequence was used to detect T_2_ relaxation time of the samples. Samples were placed in the center of the RF coil, and the FID signal was used for CPMG pulse sequence scanning [19]. CPMG detection parameters were set as the following: SW (interval between points) = 200 kHz; Tw (waiting time) = 2500 ms; NECH (no. of points of determination) = 8000. Transverse relaxation patterns were obtained using NMR relaxation time inversion fitting software.

Pseudo-color images (in BMP format) for the water absorption pattern of lily bulb scales were illustrated by NMImaging software [20], and the parameters were: FOV read = 100 mm × 100 mm; Slices = 1 mm; Slice width = 9 mm; Read size = 256; Phase size = 192; Average = 6; TE (two peaks interval) = 20 ms.

### 2.5. Morphology Observation by Scanning Electron Microscopy

The back scattered electron detector of the SEM was used to capture morphology images of the dried lily scales and starch from each of them at the accelerating voltage of 5 kV. Each sample was fixed to a slab with double-sided conductive glue, sprayed with a gold coat, and then placed in the chamber of desktop scanning electron microscope (SEM, Phenom XL, Phenom, Eindhoven, Netherlands). Images for the lily scale’s surface or cross section were obtained in magnification of 330× or 1100× and starch images in magnification of 500× [21].

### 2.6. Micro X-ray Computer Tomography (CT) Imaging

The micro X-ray CT images of each dried lily scale were collected using a benchtop X-ray CT scanner (NanoVoxel-3000, Tianjin Sanying Precision Instrument Co., LTD, Tianjin, China) at a voltage of 30 kV and a current of 100 μA; in addition, the pixel size of the detector was 49.5 μm. One piece of lily scale sample (5.0 mm × 5.0 mm size) was placed in the center of the turntable with source object distance being 11.74 mm and source detector distance 270.60 mm; a total of 1440 images (in the RAW format) at 2.0 µm resolution were recorded in one rotation with exposure time being 0.8 s. After scanning and reconstructing, approximately 2000 original CT image slices were obtained from each sample across the longitudinal (X–Y plane slice) axis [22]. 

### 2.7. Lily Starch Isolation

Alkali steeping method [8] was used for extraction of lily starch. Lily bulb scales mixed with an appropriate amount of cold distilled water were milled for 2 min, and the obtained pulp was then filtered against 100-mesh nylon screen. The material retained in the sieves was milled and sieved again four additional times. All filtrate was merged and centrifuged at 5000× *g* for 10 min at 15 °C, and precipitate was harvested. In order to further remove the residual protein, the obtained precipitate was mixed with 0.05 mol/L NaOH solution in a ratio of 1:4 (*w*/*v*, g/mL), kept at 5 °C for 10 h, and stirred every 1 h. The mixture was then centrifuged, and the precipitate was collected. The above treatment was repeated until the supernatant became transparent. The starch samples were freeze-dried, sieved through 100 mesh, and stored in a dark container kept in a desiccator awaiting further analysis. The hot air dried, microwave vacuum dried, vacuum freeze dried, and untreated lily starch samples were named as HADS, MVDS, VFDS, and FS, respectively.

### 2.8. X-ray Diffraction

Crystalline structure of lily starch was analyzed by an X-ray diffractometer (Ultima IV, Rigaku Corporation, Akishima-shi, Tokyo, Japan) according to Pozo [23]. All starch samples were scanned in the 2θ angle range of 3°~45°, with a step size of 0.02° (2θ) and scan speed of 2°/min, using an X-ray radiation source of Cu-Kα at 40 kV and 30 mA. The relative crystallinity was calculated as the ratio of the area of crystalline and amorphous regions on the X-ray diffractograms using JADE 6.0 software (Material Data Corporation, Livermore, CA, USA).

### 2.9. Pasting Profiles Determination 

Pasting properties of lily starch were determined by a Rapid Visco Analyser (RVA-4; Newport Scientific Pt. Ltd., Warriewood, Australia) according to Ziegler [24]. In an aluminum test tube, 3.00 g of lily starch and 25.0 mL of deionized water were combined. The slurry was elevated to 95 °C for 2.5 min and held at that temperature for 5 min before being cooled to 50 °C in 4 min and equilibrated for 2 min. Starch pasting profiles were obtained, and peak viscosity (PV), breakdown (BD, which is PV minus TV), final viscosity (FV), setback (SB, which is FV minus TV), peak time (Pt), and pasting temperature (PT) were obtained from the pasting curves by using the “Thermocline software” provided with the instrument.

### 2.10. Solubility and Swelling Power

Solubility and swelling power of lily starch were determined according to Waterschoot [25]. An amount of 0.300 g lily starch was mixed with 30.0 mL distilled water evenly and heated in a water bath at 50 °C for 30 min and then cooled to ambient temperature. Precipitates obtained by centrifugation (3000× *g*, 20 min) were weighed, and the resulting supernatants were dried at 105 °C until the weight remained constant. The solubility and swelling power were calculated as Equation (3):

S(%) = A/W × 100%; SP(g⁄(g) = P/(W × (1 − S)).(3)

where S is the solubility of starch; SP is the swelling power of starch; A is the constant quality of the supernatant after drying (g); W is the weight of the starch sample (g); P is the mass of precipitated substance after centrifugation (g).

### 2.11. Statistical Analysis

Six parallel samples were measured in each experiment, and results were expressed as mean value ± standard deviation (*p* < 0.05). Experimental results were plotted using Origin Pro 2021 software (Origin Lab, Inc., Northampton, MA, USA), and data analysis and ANOVA were performed by using SPSS 26.0 software (SSPS, Chicago, IL, USA).

## 3. Results

### 3.1. Rehydration Performance 

**Figure 1:**
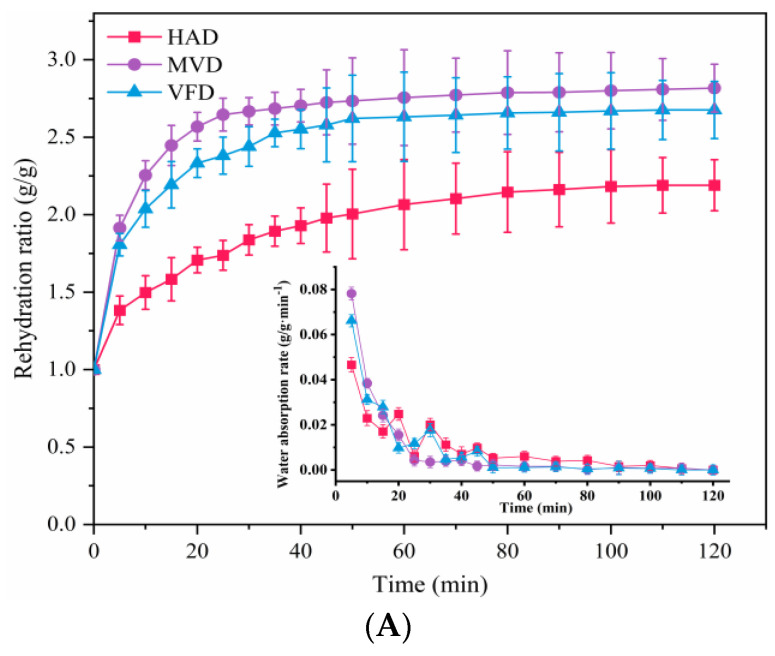

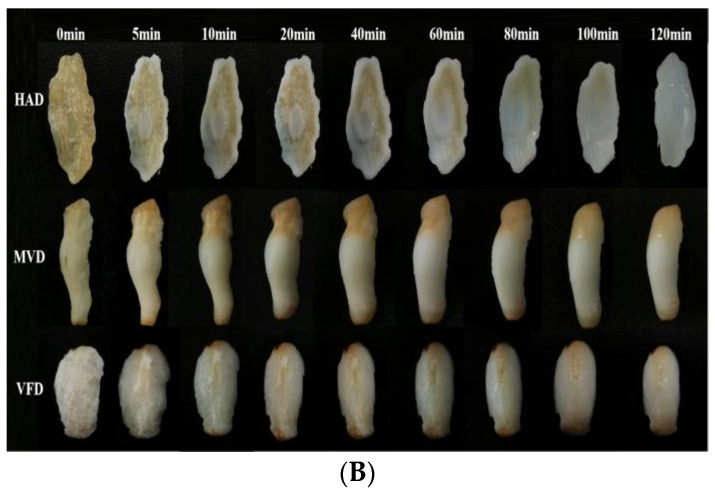
Rehydration performance of dried Lanzhou lily scales: (**A**) rehydration curve. (**B**) photos of lily scales at the set time interval during rehydration (0 min–120 min). HAD: hot air drying, MVD: microwave vacuum drying, VFD: vacuum freeze drying. Each value is expressed as mean ± S.D. (*n* = 3).

Rehydration capacity of dried lily scale samples obtained by HAD, MVD, and VFD is shown in Figure 1. Rehydration kinetic curves, coupled with water absorption rate profiles (Figure 1A), showed the same variation tendency, in which the rehydration ratio increased rapidly in the initial stages, then slowed down, and eventually went into plateau. These were in accordance with the results of Kocabay and Ismail [26], and a similar process was also observed in dried pineapple rehydration by Kumar [27]. Moreover, it deserves attention that among these three dried lily scale samples, the moisture content of the MVD sample was always the highest and almost reached the equilibrium at 60 min, while that of the VFD sample and HAD sample reached the equilibrium at 80 min and 110 min, respectively. The MVD sample almost possessed the highest rehydration rate in the first 20 min. Photos (Figure 1B) illustrated the rehydration state of the dried lily scale at different intervals during a 120-min period: lily scales from each group had different shapes and colors; the unsoaked sample of HAD appeared flat and more yellowish-brown, and when it absorbed water, the color of the rehydration area gradually became shell-like white, and that of the anhydrated area remained a little yellowish-brown; the unsoaked sample of VFD also appeared flat but whiter than HAD, and when it soaked up water, the color of it gradually became a little jade-like white during a 120-min period; the unsoaked sample of MVD appeared more cylindrical, which might be the result of rapidly dehydrating during the drying process and subsequently curling, and its color showed a little more yellowish-brown, which ought to be a result of microwave heat effects, and when it soaked water the color of it became more jade-like white. The rehydration tendency results shown in Figure 1B were consistent with those of Figure 1A. Therefore, it was simple to figure out that, among these three dried samples, the MVD sample had the quickest rehydration rates to attain stable moisture content. The brown color of the MVD lily scale tip was attributed to the greater energy input to the lily scale tip area during MVD treatment, which resulted in the pasting phenomenon, which is another intriguing occurrence observed in Figure 1B. At the end of the 120-min rehydration, all three samples still had a yellowish-brown section, more or less, and all samples might have reached the water absorption limit.

### 3.2. Water Migration and Distribution within Lily Scales during Rehydration

**Figure 2:**
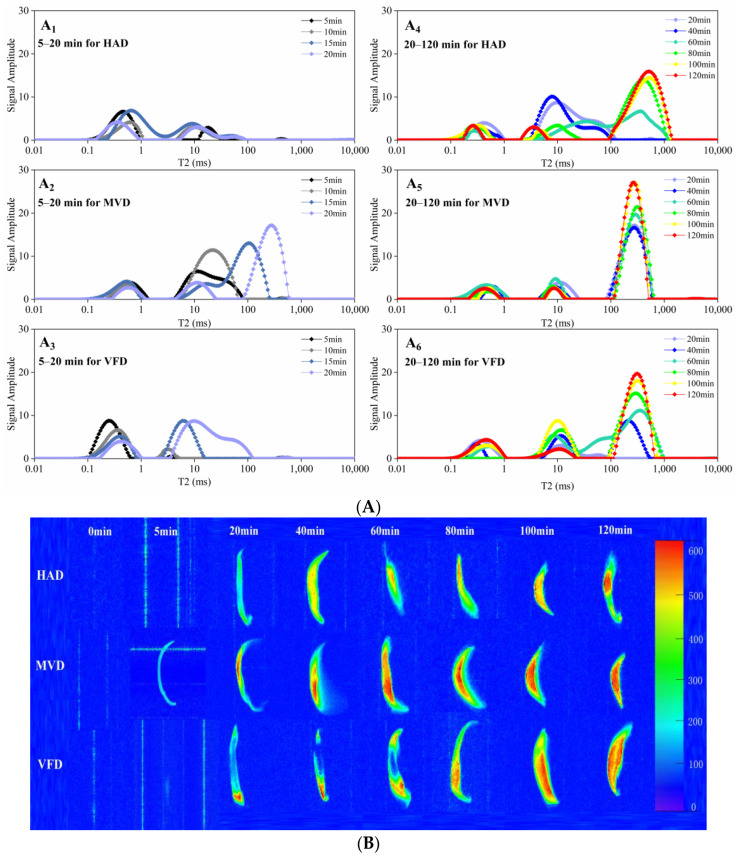
Water migration and distribution during the rehydration process (0–120 min). (**A**) Distribution of T_2_ relaxation times. (**B**) pseudo-color images of water migration during rehydration, the colors (blue through green and yellow to red) represent the increasing moisture contents in lily scale.

In order to further understand the rehydration behavior of dried lily in detail, water migration and distribution in lily scales during rehydration were illustrated by LF-NMR, which is characterized by transverse relaxation time (T_2_) and a quick, potent, and non-destructive examination method for recording water distribution or migration within foodstuffs [28]. Usually, three relaxation proton components, T_21_ (0.1–1 ms), T_22_ (1–100 ms), and T_23_ (100–1000 ms), represent the bound water (the lowest mobility water), the relatively weakly bound water (also called immobilized water), and the free-state water [29], respectively. First, it was simple to see that rehydration caused the T_2_ components (T_21_, T_22_, and T_23_) of all groups to shift to a longer relaxation time, demonstrating that all three water populations within each lily scale became more mobile as rehydration processing continued; second, there appeared to be different water migration profiles among the three sample groups, not only in the interval of 5–20 min (Figure 2A(A_1_–A_3_)) but also in the interval of 20–120 min. (Figure 2A(A_4_–A_6_)). At the 5 min interval: the HAD group showed a larger A_21_ peak area and a very small A_22_; the VFD group showed a larger A_21_ and a little small A_22_; the MVD group showed a little small A_21_ and larger A_22_. This could be the result of the dried lily scales being extremely dehydrated at this time, as all three groups were showing almost no A_23_ area at this time. At the 10–20 min interval: A_22_ became larger in all groups, and A_23_ (free water population) began to increase and become the largest part at 15 min and 20 min in the MVD group, which showed that restored water began to appear more mobile, especially in the MVD group, and it was the easiest for water ingression in MVD and the hardest in HAD. At 20–120 min intervals, A_21_ and A_22_ of MVD nearly remained unchanged; however, A_23_ of it grew larger and larger; A_21_ and A_22_ of HAD significantly varied, and A_23_ appeared at 60 min and then grew; A_21_ and A_22_ of VFD varied moderately, and A_23_ appeared at 40 min and then grew; however, A_23_ areas of all groups at 100 min and 120 min were nearly coincident, indicating that rehydration capacities for lily scales of all groups came to a level-off stage at this time. Results of Figure 2A show a noticeable difference in rehydration capacities and degree of difficulty among all dried lily scale groups; the MVD sample had the highest rehydration rate, the VFD sample was moderate, and HAD sample was the lowest.

Pseudo-color images produced by “NMImaging software” can effectively show how water is distributed or migrates through different foodstuffs or materials. Typically, a higher proton signal density (namely higher moisture content) is marked in redness, and a lower one (namely lower moisture content) is marked in blueness [30]. Rehydration profiles expressed in pseudo-color images in Figure 2B exhibited that: (1) At 0 min and 5 min, water contents within three groups were so low that proton signals could not be seen on the blue background, except that the signal of MVD at 5 min could be visualized. (2) At 20 min and 40 min, more water gradually filtered into the lily scale matrix so that proton signals for all samples appeared. A significant amount more water had already entered the nearly central portion of the MVD, while only a small amount of more water had crowded the tip of the VFD, suggesting that the water distribution there was uneven. In addition, less water had entered the HAD sample at this point, and the water distribution there appeared to be relatively more uniform. (3) Around 60 to 120 min, the moisture content practically reached its peak, water absorption leveled off, and its arrangement became more even, as seen by variations in redness, yellowness, and greenness; the order of water distribution uniformity was MVD > VFD > HAD. In brief, it was discovered that MVD samples had the best rehydration ability and that their water absorption reached equilibrium first. Additionally, MVD samples’ water distribution was the most uniform. Both of these findings were consistent with the results of previous studies on transverse relaxation patterns.

### 3.3. Morphology and Microstructure of Dried Lily Scale

**Figure 3:**
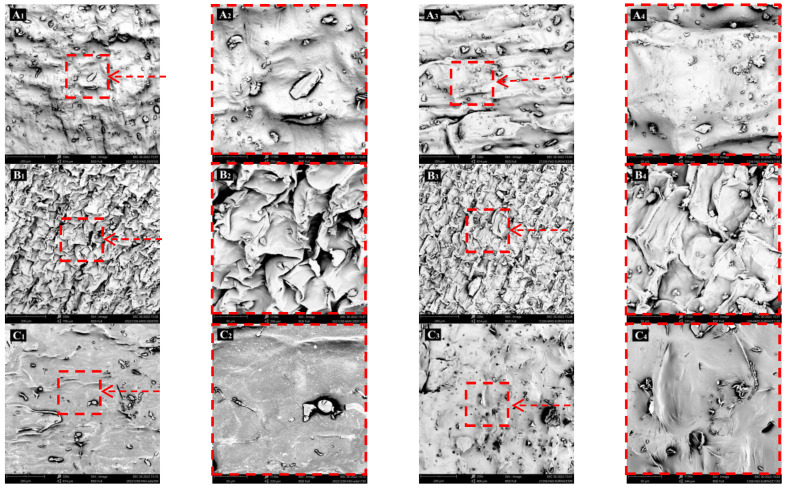
SEM morphology for dried lily scales. (**A_1_**,**A_2_**), (**B_1_**,**B_2_**) and (**C_1_**,**C_2_**) are surface micrograph for HAD, MVD, and VFD lily scales at 330×/1150×, respectively. (**A_3_**,**A_4_**), (**B_3_**,**B_4_**) and (**C_3_**,**C_4_**) are cross-section plane micrographs for HAD, MVD, and VFD lily scales at 330×/1150×, respectively. (**A_2_**) is the magnification of the red dotted box in (**A_1_**), (**A_4_**) is the magnification of the red dotted box in (**A_3_**), others are the same as above.

The differences in structure and porosity found after drying could be the cause of different rehydration performances. It was crucial to further identify the diversity of morphology and microstructure in dried lily scale samples from the three groups in order to understand why there were varied rehydration performances among dried lily scale of HAD, MVD, and VFD. Each sample group’s morphological characteristics were recorded by a scanning electron microscope (SEM) at a magnification of 330/1100, and the results are shown in Figure 3. It could be clearly found that: both the surface and the cross-section of HAD samples (Figure 3A_1_–A_4_) looked relatively rougher and a little more wrinkled. There unevenly existed plenty of tiny/small bulges with difference sizes and shapes, only a small number of pores were present on the surface (Figure 3A_1_,A_2_), and few channels in the cross-section (Figure 3A_3_,A_4_); the VFD sample (Figure 3C_1_–C_4_) showed as more smooth and less wrinkled, more pores in the surface (Figure 3C_1_,C_2_), and more channels in the cross-section (Figure 3C_3_,C_4_); MVD samples (Figure 3B_1_–B_4_) had much more intensive wrinkles in a certain order or showed scale-like on the whole, much more pores present in the surface (Figure 3B_1_,B_2_), and much more channels in the cross-section (Figure 3B_3_,B_4_), and those possibly were the results of intensive thermal effects of microwave and boiling effects under vacuum conditions. Therefore, it could be found that the more porous and wrinkled network structure (both surface and inner) of MVD samples was one of the reasons responsible for its highest water absorption performance and also the good performance of VFD samples [31].

**Figure 4:**
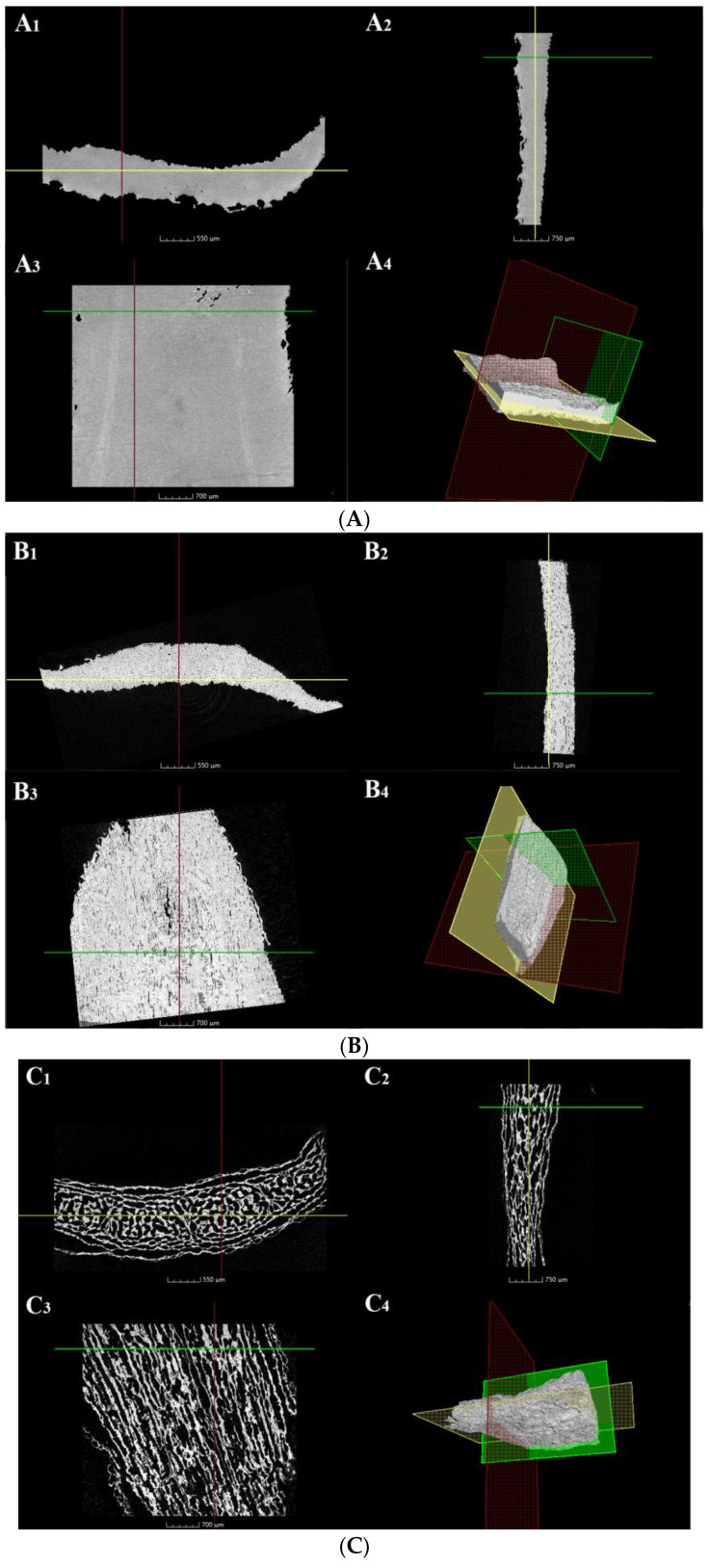
Micro X-ray computed tomography (CT) images of dried lily scales. (**A**–**C**) are the CT images for HAD, MVD, and VFD, respectively; (**A_4_**,**B_4_**,**C_4_**) are the three-dimensional CT images; (**A_1_**–**A_3_**), (**B_1_**–**B_3_**), and (**C_1_**–**C_3_**) are CT images of perpendicular slice; those colored planes or lines at images indicate each slice location.

Micro X-ray CT is a powerful non-destructive imaging technique, which can show the internal spatial structure of tested samples in detail; in particular, it is very effective for illustrating the porous inner network structure of materials, since air voids were clearly marked off from the solid phase [32]. Figure 4 reveals the internal spatial structure of lily scale from three sample groups. It was obvious that: (1) the structure of HAD samples (Figure 4A(A_1_–A_4_)) was impacted with almost no pores and channels, which was consistent with the result of Aravindakshan [33]. It was confident that high temperature (at 80 °C) during HAD could cause structure collapse of dried foodstuff and result in closed pores and a dense/shrunken internal structure; another reason might be that starch pasting induced by heating at 80 °C could make starch molecules attach with each other, which resulted in a whole compact structure. All these eventually led to the relatively worse rehydration performance of HAD samples; (2) the structure of MVD samples (Figure 4B(B_1_–B_4_)) showed as more porous and looser with many more channels in a much more porous structure. The research of Zhou [17] indicated that MVD, the combination of positive vapor pressure produced by vacuum and efficient internal heating effects of microwave, could easily create “channels” in porous structures in dried materials; (3) VFD samples (Figure 4C(C_1_–C_4_)) possessed the loosest structure with more channels in the largest diameter and highest porosity. It had already been proven that VFD can create relatively larger pores in food matrix because of ice crystals’ formation during the freezing process, and the very high vacuum condition also can help form a highly porous structure in dried materials [31]. It was evident that these formed channels in dried lily scale matrix (both MVD and VFD) could connect pores on the surface to the central parts and allow the whole matrix of lily scale to be accessible to water molecules from the external environment during rehydration. Therefore, higher porosity with more pores and more channels was one of the reasons why VFD and MVD samples had better rehydration performance.

There was also an intriguing paradoxical phenomenon: the dried lily scale of MVD clearly had lesser porosity and narrower pathways linking its matrix to the outside world than that of VFD. The water distribution in the rehydrated MVD lily scale, however, appeared to be much more even than that of the VFD sample, and the MVD sample exhibited a superior capacity for rehydration than the VFD one. The research of Zhou [31] deemed that the presence of “air bubbles” (occupying pores, cavity, and channels in dried food matrix) in the VFD sample would prevent the entry of water during rehydration, resulting in relatively poor rehydration performance of VFD dried lily scale. Nevertheless, the MVD sample also had pore, cavity, and channel in moderate size and ought to possess “air bubbles” too, while these seemed to have no impact on rehydration of the MVD lily scale. Consequently, there ought to be other contributory factors that brought about rehydration variations between VFD and MVD. One evident fact is that starch is the most abundant among those macro-components in dried lily scale. As described before (Section 2.2), the HAD sample was operated at 80 °C for 4 h, and MVD processing combines vacuum conditions with microwave energy, which can bring about heating or thermal effects to components of foodstuff during drying. Both of them could cause starch pasting or partly pasting. However, VFD is a kind of non-thermal drying technology, and thus there was no thermal effect on starch to cause pasting during drying. It is known that the solubility and hydrophilicity will increase after its pasting. Thus, we assumed that starch pasting or partly pasting caused rehydration differences among these three kinds of dried lily scales. In order to prove this assumption, lily starch isolation was carried out for further evaluation.

### 3.4. Starch Morphology by SEM

**Figure 5:**
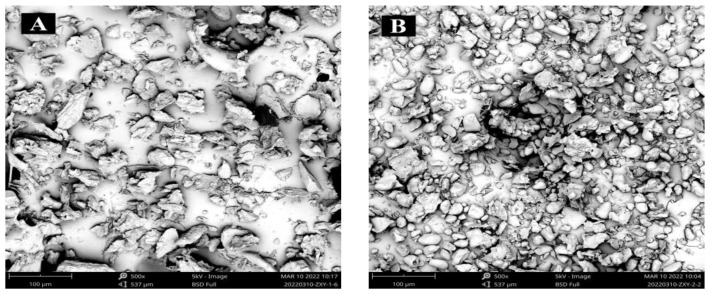

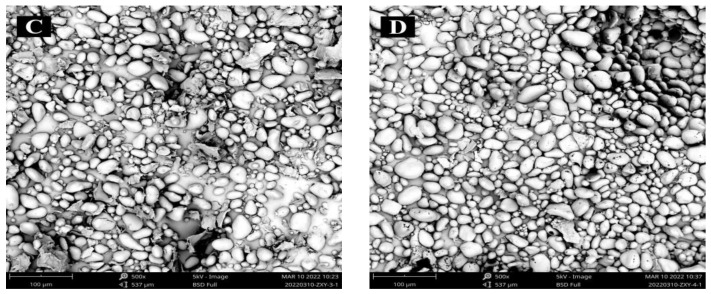
Morphological characteristics of the lily starch granules under SEM. (**A**–**D**) are micrographs (at 500×) of starch granules isolated from HAD, MVD, VFD, and fresh lily scale, respectively.

SEM is also a common way to illustrate the morphological characters of starch granules, such as shape, size, and degree of surface roughness. Normally under SEM observation, fresh lily starch granules have a smooth surface and show an elliptical or round shape, with the X-ray diffraction pattern of them being the typical B-type, the same as that of potato starch [34], and the size of starch granules ranged from 5.26 μm to 116.09 μm [7]. Morphology images (×500) of isolated starch granules are shown in Figure 5: (1) The majority of FS granules (Figure 5D) had a smooth surface and oblong-oval shapes, and a small number of FS granules had nearly triangular and spherical shapes, which coincided with the research of Yu [7]; (2) VFDS granules remained almost intact compared with FS granules; the surface appeared basically smooth and the shape looked the same as those of FS, which indicated that starch granules received little damage under the treatment of VFD; (3) Although a few MVDS granules had the relatively smooth surface and almost the same shape as those of FS, most of the granules had obvious cracking, crazing, and crushing on the surface; the surface depression of some starch granules could be intensified, and even some flakes came into being, all of which showed that lily starch granules underwent much more damage during the treatment of MVD. The possible reason is that during MVD processing, polar water molecules in food matrix absorb microwave energy, which can be continuously converted into thermo-type energy to form a vapor pressure to a certain extent. When the pressure reaches or exceeds the strength that the molecular chain organization structure in starch granules can withstand, starch granules will break, and subsequently the granules’ morphological appearance and internal texture will change, and even some of the starch granules were partly gelatinized and merged with each other into some flakes [35]. (4) Uneven granule surface features, including some pits, fissures, and folds, the presence of flake or flake-like blocks, and uniform larger blocks that may have resulted from agglomeration following starch pasting suggested that HADS granules seemed damaged and fluctuated wildly throughout drying. This result was also shown by the research of Li [36]. HAD caused starch pasting, which was followed by aggregation. Thermal, pressure, and water vapor energy all had a role in the creation of the damaged structure. In short, different drying techniques, such as HAD, MVD, and VFD, may cause varying degrees of damage to lily starch granules. The reason could be that whereas VFD would not cause partial starch gelatinization, HAD and MVD might.

### 3.5. X-ray Diffraction Patterns of Lily Starch

**Figure 6:**
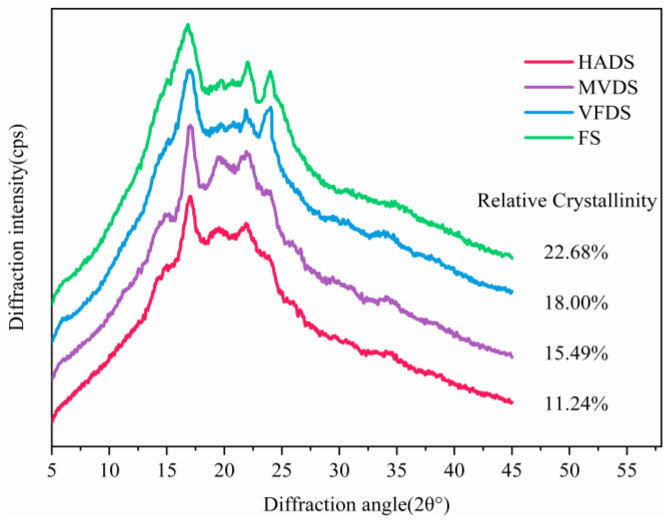
X-ray diffraction patterns and relative crystallinity of starch from each of the dried lily scales (HAD, MVD, and VFD) and fresh lily scales.

In order to ascertain effects of drying methods on lily starch molecules, the crystalline characterization of isolated lily starch was investigated by X-ray diffraction. Obtained XRD patterns and relative crystallinities for each starch were shown in Figure 6. FS exhibited typical B-type semi-crystalline diffraction patterns characterized by a small peak at 2θ of 5.6°, and a strong peak at 2θ of 17°, accompanied with double adjacent shoulder peaks at 2θ of 22° and 24° [34]. The diffraction pattern of VFDS was nearly identical to that of FS, and there was only a tiny difference in the exact position of the double adjacent shoulder peaks at 2θ of 21.84° and 24.03°, indicating that VFDS had essentially no damage during drying. On the other hand, although XRD patterns of MVDS and HADS maintained the strongest peak at 2θ of 17°, and one of the double adjacent shoulder peaks at 2θ of 24°, there were yet relatively bigger changes happening to them, in which the small peak at 2θ of 5.6° disappeared, the X-ray intensity in peak around at 2θ of 24° deceased a lot or even almost disappeared (in HADS), and even an obvious peak around 2θ of 20° came into being. Those changes in the diffraction pattern of starches could be attributed to thermal effect during HAD and MVD dehydration treatment, which could disrupt starch crystallites and/or change the crystalline orientation. Additionally, the diffraction peak around 2θ of 20° is one of the typical features for V-type starch crystallites, which commonly exist in starch isolated from cooked foodstuff (such as cereals), and are the result of the usual interaction between amylose and lipids [37], indicating that HADS and MVDS might both be partly pasted during dehydration [38]. Relative crystallinities for each starch sample in descending order were: FS (22.68%) > VFDS (18.00%) > MVDS (15.49%) > HADS (11.24%). This also confirmed that MVDS and HADS had undergone thermal treatment and had been partly pasted. Although microwave had a higher energy intensity than hot air heating, the duration of MVD was shorter than HAD because MVD was performed under the help of vacuum conditions, and the moisture evaporation rate was very fast; as a result, the starch damage created by MVD was much lower than that by HAD. Regardless, each of MVD and HAD still might be more favorable for V-type crystal [39].

### 3.6. Pasting Profile

**Figure 7:**
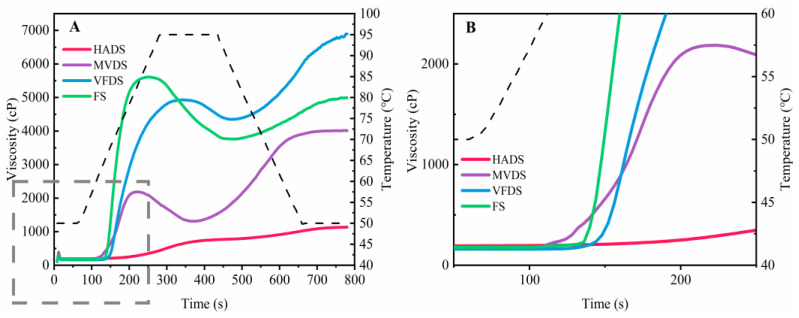
Pasting profile of starch isolated from each dried lily scale group (HAD, MVD, and VFD) and fresh lily scales. (**A**) is the whole process for pasting profile; (**B**) is the magnification of the gray dotted box (0–250 s) in (**A**).

The pasting properties of starch and starch-containing foodstuffs are normally evaluated using a Rapid Visco Analyser (RVA), in which flour–water mixture is subjected to a heat-hold-cool-hold temperature cycle to simulate the whole cooking process [40]. As initially heated, starch granules reversibly absorb water and expand, and later, when the heating process continues, the double helix crystal structure in starch begins to disappear, which is called gelatinization [41]. The viscosity of the starch paste reaches its highest when the starch particles swell to the maximum, and if at this time the heating continues, these particles would break, and the viscosity decreases [42]. Figure 7 depicts the pasting profiles of lily starch from each drying group, and Table 1 lists the pasting parameters PV, TV, BD, FV, SB, and PT. PT is the lowest temperature needed for cooking and also for making starches that begin to gelatinize, and the higher PT suggests higher resistance of starch granules to rupture during pasting. The early changes (Figure 7B) demonstrated that the MVDS viscosity increased first, and the PT of MVDS (62.03 °C) was not only lower than the VFDS (65.02 °C), but also significantly lower than the FS (65.97 °C), indicating that the MVDS had a higher affinity than the VFDS and FS. According to the research of Xu [43], the relative crystallinity of partially gelatinized starch was negatively linked with its degree of gelatinization, leading to the conclusion that MVDS may have a higher degree of gelatinization than VFDS. Additionally, PT of HADS was not detectable since it had already been highly gelatinized by the time lily scales were exposed to HAD (at 80 °C for 4 h), as seen by the violent disintegration of starch granules (confirmed by SEM results in Figure 5). Furthermore, from Figure 7 and Table 1, it also obviously could be found that drying methods showed significantly effects on PV of lily starches (*p* < 0.05), and the descending order of PV was: FS (5973.0 ± 88.84 cp) > VFDS (5097.33 ± 63.07 cp) > MVDS (2186.60 ± 7.77 cp) > HADS (624.67 ± 27.47 cp), and the PV of MVDS and HADS decreased by 57% and 88% compared to VFDS samples, respectively. PV is the highest viscosity of starch during gelatinization [44] and represents the starch’s performance and granule integrity as well as the resistance of swelling granules to shear. Typically, the crystallinity and rigidity of the granules were the key influences on pasting features, especially PV and PT [45]. The crystallinities of MVDS and HADS were significantly lower than that of FS and VFDS (shown in Figure 6).

### 3.7. Solubility and Swelling Power

**Figure 8:**
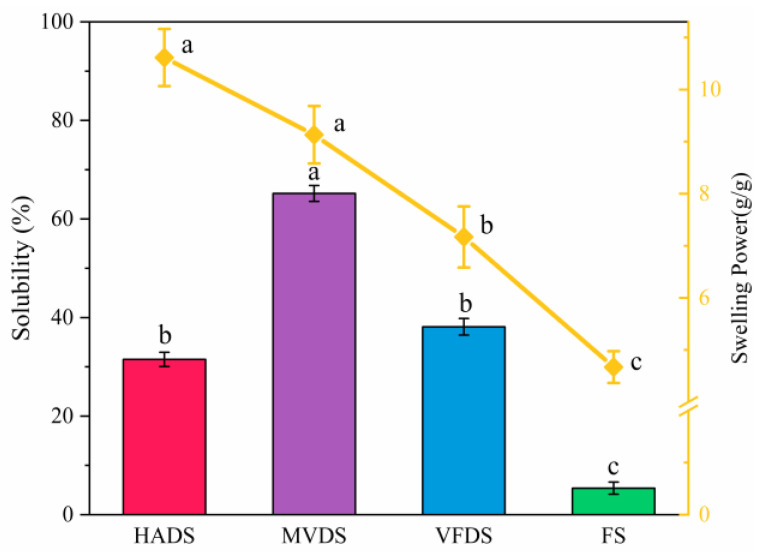
Solubility and swelling power of starch isolated from each of the dried lily scales (HAD, MVD, and VFD) and fresh lily scales. Each value is expressed as mean ± S.D. (*n* = 3), according to Waller–Duncan’s range test (*p* < 0.05); different letters in the same items indicate a significant difference.

To better understand the solubility and swelling power of dried lily scales obtained by various drying methods at 50 °C, as well as the potential effects of starch’s water-binding ability and water affinity on the rehydration performance of lily scales, these measurements were made. The results are shown in Figure 8. Under the action of heat absorption (at 50 °C) and water-binding, starch granules swelled gradually, together with the breakage of hydrogen bonds, and the transformation of the crystalline region into an amorphous region [8]. The swelling powers in descending order were: HADS (10.62 ± 0.55) > MVDS (9.13 ± 0.54) > VFDS (7.17 ± 0.59) > FS (4.67 ± 0.31), indicating that drying could significantly affect the swelling power of starch (*p* < 0.05). The lower swelling capacity represents the stronger bonding ability among starch molecules, which was in agreement with the previous results of Liu [46]. However, the highest solubility was observed in MVDS (65.19 ± 1.60) and the solubility of VFDS and HADS were 38.13 ± 1.69 and 31.51 ± 1.44, respectively. It is well-known that solubility and swelling power are important properties of starch. The original semi-crystalline structure of starch granules was damaged, in part due to breakage of intermolecular hydrogen bonds during gelatinization. Therefore, water molecules can easily diffuse into starch granules and form new hydrogen bonds with exposed hydroxyl groups from starch molecules. In the meanwhile, the mobility of starch molecules and the leaching of amylose increased, which increased the solubility and swelling power of starch. The results of solubility and swelling power of lily starches, as well as the results of pasting properties (Section 3.6), conclusively demonstrated that MVDS had higher water binding or water holding capacity than VFDS, which may explain why dried lily scales of MVD had excellent rehydration performance compared to that of VFD.

## 4. Conclusions

This study examined the effect of various drying methods on the rehydration capacity of dried Lanzhou lily bulbs. The results show that MVD and VFD can both rapidly increase the number of “channels” in the porous structure, leading to higher rehydration performance than HAD samples. Extraction of lily starch revealed that the HADS and MVDS granules’ crystallinity was reduced after heat treatment, with the MVDS exhibiting moderate partial gelatinization and a change from a B-type to a V-type crystal type; meanwhile, the VFDS granules exhibited similar physicochemical characteristics to the FS granules with less granule damage. Moreover, MVDS granules have a higher capability for absorbing water than VFDS granules due to their increased solubility and swelling power. Hence, despite having smaller pores and narrower “channels” than VFD, the MVD-treated dried lily scale has a higher capacity for rehydrating.

## Figures and Tables

**Table 1:** Pasting characteristics of lily starch.

Parameters	HADS	MVDS	VFDS	FS
PV(cp)	624.67 ± 27.47 ^d^	2186.60 ± 7.77 ^c^	5097.33 ± 63.07 ^b^	5973.00 ± 88.84 ^a^
TV(cp)	536.00 ± 30.33 ^d^	1313.00 ± 36.51 ^c^	4279.00 ± 74.20 ^a^	3685.00 ± 38.69 ^b^
BD(cp)	84.67 ± 10.50 ^c^	856.32 ± 18.74 ^b^	788.00 ± 52.61 ^b^	2318.67 ± 60.00 ^a^
FV(cp)	1120.00 ± 63.79 ^d^	3997.68 ± 30.99 ^c^	6932.33 ± 59.43 ^a^	4981.67 ± 78.56 ^b^
SB(cp)	461.33 ± 6.86 ^c^	2680.87 ± 12.11 ^a^	2568.33 ± 15.50 ^ab^	1298.00 ± 10.33 ^b^
PT(°C)	ND	62.03 ± 4.30 ^b^	65.02 ± 7.23 ^ab^	65.97 ± 6.66 ^a^

Note: means in the same column with different lower-case letters are significantly different (*p* < 0.05). ND: not detected.

## Data Availability

Data will be made available on request.
